# Accelerating Characterization of Therapeutic Antibodies: A Comparative Assessment of icIEF-UV/MS and the Traditional Fractionation Workflow

**DOI:** 10.1021/jasms.5c00058

**Published:** 2025-07-28

**Authors:** Arnik Shah, Parth Shah, Alex Johnson, Jean Bender, Dmitry Gumerov, Jingwen Ding, Scott Mack, Matthew D. Stone, Maggie A. Ostrowski

**Affiliations:** † 647828 Visterra Inc, 275 second avenue, Waltham, Massachusetts 02451, United States; ‡ 266634 SCIEX, Fremont, California 94538, United States

**Keywords:** Charge variant characterization, Biologics, Imaged capillary isoelectric focusing (icIEF), Mass spectrometry (MS), Post-translational modifications (PTMs), Biologic licensing application

## Abstract

Accurate characterization of charge variants in biologics is crucial to uphold stringent quality standards ensuring the safety and efficacy of biotherapeutic products and regulatory requirements for the Biologic Licensing Application (BLA) process enabling marketing application. These variants, arising from post-translational modifications (PTMs) during upstream processing and enzymatic/nonenzymatic reactions in downstream processing and storage, can significantly impact therapeutic potency, efficacy, and immunogenicity. Conventional methods for characterizing charge variants typically involve labor-intensive fraction enrichment, consuming time and resources. However, recent technological advancements, exemplified by the Intabio ZT system’s innovative platform, enable the seamless and direct integration of imaged capillary isoelectric focusing (icIEF) and UV quantitation with mass spectrometry (MS) PTM identification, facilitating rapid and unbiased characterization. In this study, we conduct a comparative assessment of the charge variant characterization of investigative mAb-1 using icIEF-UV/MS analysis and a traditional fractionation-based workflow. Our results demonstrated that icIEF-UV/MS-based proteoform characterization provided comparable icIEF-UV separation and quantitation and superior direct MS-based peak characterization with shorter time and less sample processing compared to conventional fractionation approaches.

## Graphical abstract

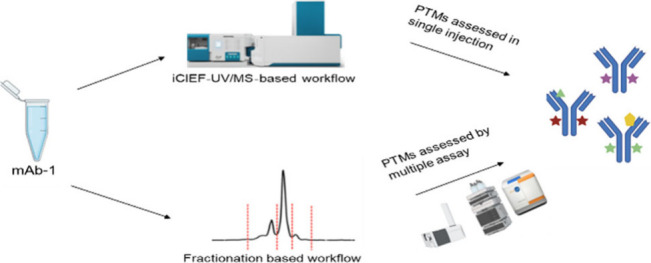

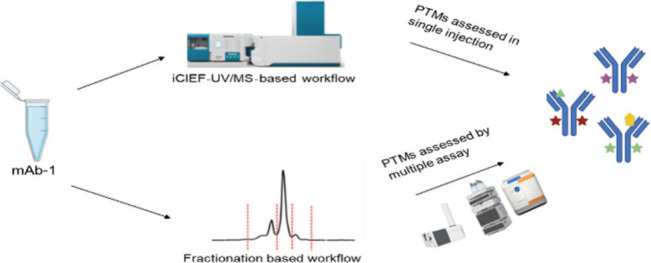

## Introduction

Monoclonal antibodies (mAbs) have emerged as a preeminent therapeutic modality, offering unprecedented specificity in targeting various diseases.^1–4^ The groundbreaking hybridoma technique, pioneered by Köhler and Milstein in 1975, revolutionized the field by enabling large-scale production of highly specific mAbs.^5–8^ Subsequent advancements in purification methodologies have significantly augmented the clinical and research potential of these biomolecules.^9,10^ Over the past quarter-century, mAbs have become the cornerstone of treatment strategies for diverse pathological conditions, with 80 therapeutic mAbs currently approved by the US FDA and numerous undergoing clinical trials.^11,12^

Post-translational modifications (PTMs) play a critical role in determining the efficacy and safety profile of biologics, particularly mAbs. These enzymatic and nonenzymatic modifications can occur during biosynthesis and purification or accrue during storage. In the context of therapeutic mAbs, common PTMs including glycosylation, disulfide bond formation, N- and C-terminal modifications, and proteolytic cleavage may arise during bioreactor processing.^13–19^ Furthermore, chemical modifications may arise during the purification process, formulation, and storage, such as oxidation, deamidation, and glycation.^18,20^ Both enzymatic and chemical modifications can profoundly influence biological activity, pharmacokinetics, and immunogenicity of biologics.^21,22^

The comprehensive characterization of these modifications is an essential component of biotherapeutic development and constitutes a critical aspect of the Biologics License Application (BLA) process as it aids in characterization of critical quality attributes (CQAs),^23–26^ justifying specification for a process enabling safe and effective therapeutic production. Consequently, the implementation of analytical methodologies is imperative for the precise characterization and quantification of these modifications, ensuring their presence within predetermined limits.^27–29^ This rigorous analytical approach is fundamental to maintaining the quality, safety, and efficacy of biologic therapeutics throughout their development and clinical application.^30,31^

Specific PTMs impart distinct charge characteristics to mAbs, significantly affecting their isoelectric points (pI).^32,33^ Imaged capillary isoelectric focusing (icIEF) effectively separates these charge variants based on their net pI difference into 3 distinct regions: acidic, main, and basic regions.^34–36^ Traditionally, the characterization of post-translational modifications (PTMs) associated with charge variants involves fraction collection, sample concentration, and subsequent mass spectrometric analysis.^37^ This workflow is often time-consuming and sample-intensive and may introduce analytical artifacts due to extensive sample handling, including multiple concentration steps and exposure to variable storage conditions such as freeze–thaw cycles. This process can lead to the characterization of PTMs that are not representative of drug product.

Significant advancements have been made in native mass spectrometry (MS) approaches for characterizing charge variants of mAbs.^38–40^ However, discrepancies can arise between liquid chromatography, as chromatographic profiles may not match with electropherograms obtained from icIEF methodologies. Moreover, recent advances have allowed capillary electrophoresis online coupling to mass spectrometer, which requires complex instrumentation that is not suitable for daily analysis.^41,42^ To address these challenges, we investigated an icIEF-UV/MS workflow developed by SCIEX, enabling the online coupling of icIEF to a mass spectrometer.^43^

We evaluated the charge variant profile of investigational mAb-1 using both the traditional fractionation-based workflow and the integrated icIEF-UV/MS approach enabled by the Intabio ZT system. While both methods yielded comparable results, the icIEF-UV/MS platform offers significant advantages in terms of analytical efficiency and accuracy. The icIEF-UV/MS system enables seamless, real-time coupling of charge separation, UV detection, and mass spectrometric analysis. This streamlined workflow reduces total analysis time, minimizes sample handling-induced artifacts, and improves overall data quality, thereby representing a substantial advancement in the characterization of charge variants in monoclonal antibodies

## Materials and Methods

### Reagents and Materials

Reagents of the highest purity were acquired for analysis. Highly purified investigational mAB-1 was obtained from Visterra Inc. laboratories. LC-grade sodium phosphate, potassium chloride, and l-arginine were purchased from Sigma-Aldrich (St. Louis, MO). pH-based ion exchange buffers were obtained from Thermo Fisher Scientific (Waltham, MA). Reagents for icIEF and reduced capillary electrophoresis were sourced from Protein Simple (San Jose, CA). Zeba Spin Desalting Columns (7K MWCO, 0.5 mL) were purchased from Thermo Fisher Scientific (Waltham, MA), and Pharmalyte carrier ampholytes with pH ranges of 5–8, 6–10, and 8–10.5 were purchased from Cytiva (Marlborough, MA). The Electrolytes and Mobilizer Kit and Intabio ZT cartridge used for Intabio ZT analysis were obtained from SCIEX (Framingham, MA). PNGase F for deglycosylation was obtained from New England Biolabs (Ipswich, MA).

### Size Exclusion Chromatography (SEC)

An Agilent UHPLC system (Lexington, MA) equipped with a binary pump and a column heater was used. Samples were analyzed on a 30 cm Waters Bioresolve SEC mAb column (200 Å, 2.5 μm particle size; Milford, MA) at room temperature with a mobile phase composed of sodium phosphate, potassium phosphate, and potassium chloride at pH 7.2. The flow rate was set to 0.4 mL/min. A 10 μg aliquot of each sample was injected, and analysis was performed over a 10 min isocratic gradient to obtain separation between aggregate/high molecular weight species (HMW), monomer/main peak, and low molecular weight species (LMW).

### LC-Based Fractionation

An Agilent UHPLC system (Lexington, MA) equipped with a binary pump, column heater, and automated fraction collector was used. Samples were injected at 4 mg and analyzed on a semipreparative ProPac WCX-10 weak cation exchange column (10 μm, 9 × 250 mm,); Thermo Fischer Scientific, Waltham, MA) at 30 °C; mobile phase A and mobile phase B were each diluted 10-fold to obtain 1× buffers at pH 5.6 and 10.2, respectively. Charge variant separation was achieved by applying a gradient with a 2% increase in mobile phase B per minute. Fractions were collected in time-based intervals of 20 s at the elution time of the charge variants. Overall 30 injections were performed to get the desired material for characterization.

### Imaged Capillary Isoelectric Focusing (icIEF)

An icIEF master mix was prepared by combining 70 μL of methylcellulose, 8 μL of Pharmalyte (pH 3 to 10), 4 μL of 500 mM l-arginine, 50 μL of urea, and 2 μL of reconstituted Maurice pI markers pH 6.1 and 10.14 each and making up the volume to 160 μL total with deionized water, followed by vortexing to mix. The mAb-1 material was diluted with deionized water to a final concentration of 2 mg/mL. To 40 μL of the mAb-1 sample was added 160 μL of the icIEF master mix to a final concentration of 400 μg/mL, and the mixture was vortexed and then centrifuged at 13,000*g* for 3 min. Approximately 160 μL of the resulting supernatant was transferred for analysis on a Maurice instrument (Biotechne, Minneapolis, MN). The mAb-1 sample was injected for 55 s, and separation was performed by focusing for 2 min at 1500 V, followed by 3000 V for 10 min. Absorbance was measured at 280 nm (0.005s exposure) along with native fluorescence at various exposure times (5, 10, 15, 20, and 30 s).

### Reduced Capillary Electrophoresis (r-CE-SDS)

To prepare a final mAb-1 concentration of 0.5 mg/mL for reduced analysis, 2.5 μL of a 10 mg/mL mAb-1 solution was combined with 37.5 μL of Maurice 1× sample buffer, 5.5 μL of deionized water, 2 μL of Maurice 25× internal standard, and 2.5 μL of 14.2 M β-mercaptoethanol. The samples were then heat-denatured at 70 °C for 10 min, followed by cooling in an ice bath for 5 min. After vortexing and centrifugation, the entire 50 μL sample was transferred to a vial for Maurice analysis. The injection was performed by applying 4600 V for 30 s, and separation was conducted at 5750 V over 30 min. Size variants of mAb-1 were detected by the absorbance at 220 nm.

### Deglycosylation and Intact Mass Analysis with LC-MS

Deglycosylation of mAb-1 was performed using PNGase F following the manufacturer’s instructions. Intact mass analysis for the fractionation-based workflow, an Agilent UPLC system (Lexington, MA) equipped with a binary pump and column heater was used and coupled online to an Agilent 6545XT mass spectrometer. Samples were analyzed using a 150 mm Waters BEH SE-UHPLC column (200 Å, 1.7 μm particle size) over an isocratic gradient with a mobile phase consisting of water, acetonitrile, and 0.1% v/v formic acid at a flow rate of 0.3 mL/min. Each sample (10 μg) was injected and analyzed over 10 min.

The MS was operated in positive ion mode for intact mass analysis, with single-stage MS spectra acquired within an *m*/*z* range of 2500–5500 in high-resolution mode (4 GHz). Source conditions were as follows: temperature set to 350 °C, drying gas flow at 13 L/min, nebulizer pressure at 38 psi, sheath gas temperature at 365 °C, capillary voltage at 5800 V, and nozzle voltage at 2000 V. Deconvolution was performed using BioConfirm software (v12.0), applying the intact mass deconvolution workflow with a maximum entropy algorithm. The deconvolution settings included a mass range of 120,000–160 000 Da, a mass step of 1 Da, a baseline factor of 7, and an *m*/*z* range of 2500–5500.

### Thermall Stress of mAb-1

mAb-1 was thermally stressed on a heat block at temperature 5 °C below its thermal melting point for 5 days.

### icIEF-UV/MS Analysis Conditions

The icIEF-UV/MS analysis was performed on an Intabio ZT system (SCIEX, Framingham, MA). The separation and mobilization in the sample channel was monitored with UV at 280 nm. The mAb-1 and thermally stressed mAb-1 sample were desalted with Zeba desalting columns. 200 μL of sample mix was prepared by mixing 400 μg/mL protein sample with 3% Pharmalyte 5–8, 3% Pharmalyte 8–10.5, 15 mM l-arginine, and pI markers. The analysis of the mAb-1 sample was initially performed without formamide, then 20% formamide was used to be comparable with urea addition in the release method and also prevent aggregates for the stressed mAb-1 sample. The sample was injected for 55 s. The focusing was performed by applying voltage between anolyte and catholyte electrodes for 1 min at 1500 V, 1 min at 3000 V, and 4.5 min at 4500 V. The chemical mobilization was performed by introducing the mobilizer at the end of the sample channel with a delta voltage of 3000 V between the anolyte and mobilizer electrodes. The ESI voltage was set at 5500 V, with a flow rate of 3 μL/min for 10 min.

ZenoTOF 7600 (SCIEX, Framingham, MA) was used for intact MS analysis with curtain gas of 25 psi and a nanocell temperature of 100 °C. TOF MS was performed in positive mode from 2000 to 6000 *m*/*z*, with a declustering potential of 190 V, collision energy of 55 V, and accumulation time of 0.5 s. The time bins to sum were set at 150 V, and the QJET RF amplitude was set at 270 V.

Biologics Explorer was used to deconvolute the MS data for analysis. The icIEF-MS profile was generated with time-resolved deconvolution. To generate the deconvoluted MS spectra for each peak in the icIEF-MS profile, ions between 2200 and 6000 *m*/*z* were deconvoluted with Max Entropy Deconvolution from 147 to 150 kDa.

## Results

### icIEF-UV/MS Analysis with Intabio ZT System

The Intabio ZT system was utilized for icIEF-UV/MS analysis of the mAb-1 sample, offering high-resolution separation and sensitive intact-level characterization of charge variants. This platform facilitated the concurrent detection of charge heterogeneity and post-translational modifications. The initial step involved establishing an icIEF-UV profile comparable to the method previously performed on the Maurice system, as depicted in Figure 1 alongside percent peak areas observed for both the methods. A strong correlation was observed between the UV profiles obtained from Maurice and the Intabio ZT system. The similarity between the Maurice release method and the Intabio ZT profile strengthens confidence in the accurate assignment of modifications to individual charge variants as the Maurice method is routinely used in quality control laboratories to monitor manufacturing consistency. Although minor pI differences were observed between the Maurice method and Intabio ZT analysis, we attribute this difference to the distinct master mix compositions used for the icIEF analysis in each method. The icIEF-MS profile appeared inverted relative to the icIEF-UV profile due to the mobilization process wherein basic variants are detected first in the MS analysis, followed by the main and acidic variants as seen in Figure 2A. To identify PTMsassociated with mAb-1, the main peak was deconvoluted, and the most intense deconvoluted signal was used as reference mass. The potential modifications were identified according to mass differences and the pI shift between each proteoform against the reference mass. All the deconvoluted masses related to reference mass was observed to be under 50 ppm when compared to theoretical masses.

**Figure 1:**
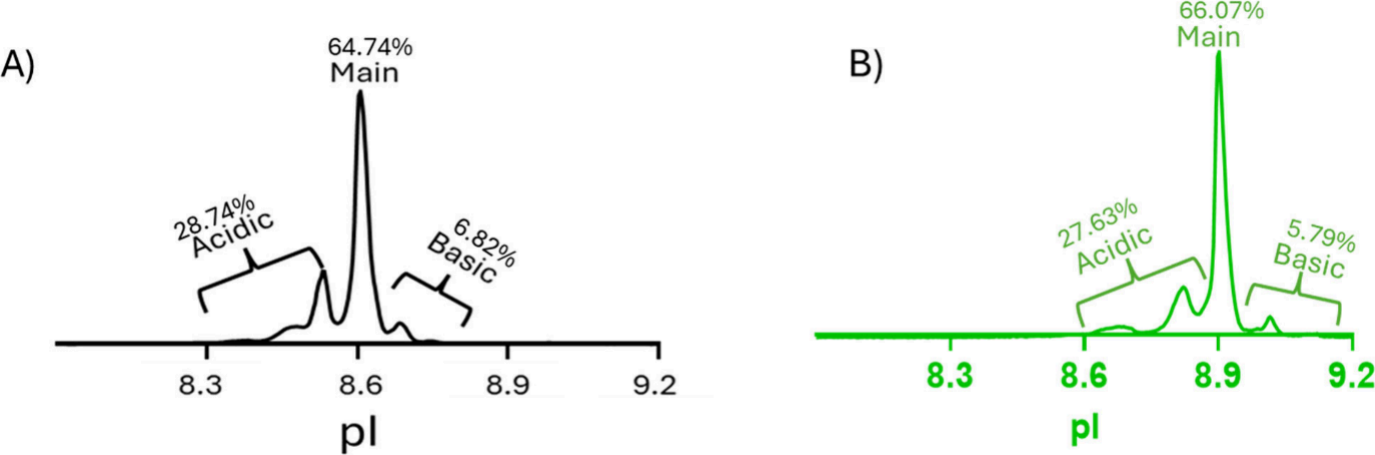
Electropherogram comparison between Maurice and the Intabio ZT system. (A) UV electropherogram from Maurice with respective % peak areas. (B) icIEF-UV profile from the Intabio ZT system with respective % peak areas.

**Figure 2:**
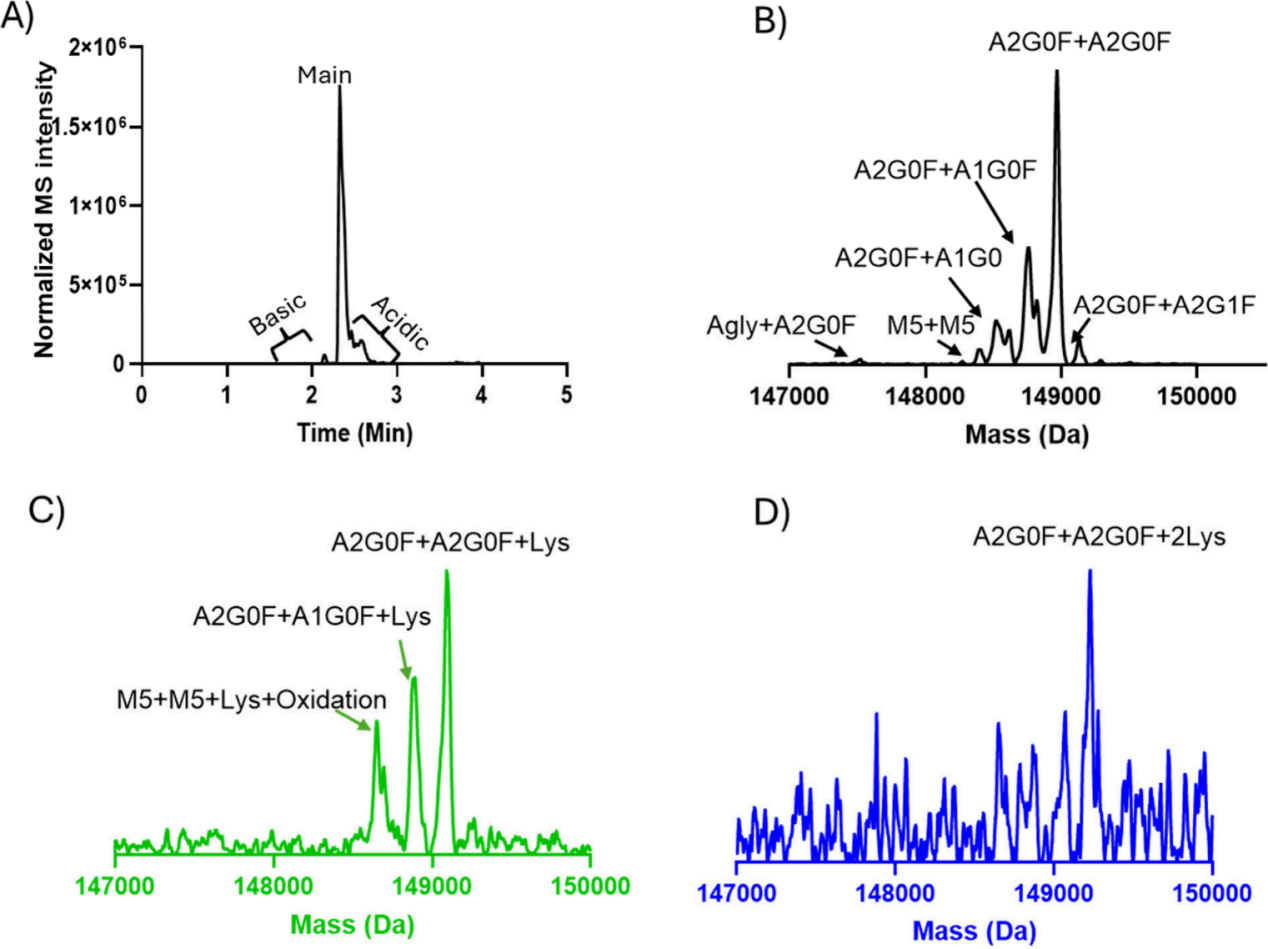
icIEF-MS profile and deconvoluted spectra of main and basic variants from icIEF-UV/MS analysis of mAb-1 with the Intabio ZT system. (A) icIEF-MS profile showing basic, main, and acidic species. (B) Deconvoluted spectrum of the main variant, displaying different glycoforms. (C) Deconvoluted mass spectrum of the basic variant, revealing similar glycoform profiles compared to the main variant with the addition of one C-terminal lysine. Additionally, potential oxidation modification was observed for high mannose species with one C-terminal lysine. (D) Deconvoluted mass spectrum of the basic variant, revealing the presence of low-abundance species of mAb-1 with two C-terminal lysine residues.

The mass spectrum related to the main peak represents the most abundant mAb-1 species, with its deconvoluted MS spectra displaying a range of glycoforms, as illustrated in Figure 2B. The main peak consists of major glycosylation pairs from A2G0F/A2G0F up to A2G1F/A2G1F, as well as aglycosylation, afucosylation, loss of GlcNAc, and high mannose species. Notably, along with the acidic shift in pI within the main peak, which is reflected by later MS detection time, the apparent relative intensity of G0F/G1F increased, as seen in Figure S1, indicating glycation as a prominent modification associated with the acidic side of the main variant. Glycation is a post-translational modification resulting from the covalent binding of a hexose to the lysine on a free amine, resulting in both an acidic shift and the addition of 162 Da.^44^ These findings suggest that the main variant consists of different glycoforms and glycation as a prominent modification, which has a slight acidic shift. This ability to detect glycated species without requiring deglycosylation treatment highlights the advantage of this 2-dimentional analysis and the instrument’s sensitivity in identifying minor modifications, eliminating the need for additional sample preparation.

Basic charge variants were identified to contain unprocessed C-terminal lysine residues, which were detected with high confidence in the MS data. These variants exhibited mass shifts of +128 and +256 Da corresponding to the addition of one or two lysine residues, designated as “Lys” and “2Lys”, respectively (as seen in Figure 2C and D). A low level of the 2Lys variant was detected, indicating its minimal presence of such modification. C-terminal lysine variants were observed across different glycosylation profiles, as shown in Figure 2C. Additionally, a mass difference of +16 Da was identified in a basic species containing high mannose with an added C-terminal lysine. This mass difference likely indicates an oxidation event on certain amino acid residues like methionine, tryptophan, and histidine, resulting in an additional mass of +15.99 Da.

The acidic variant displayed 2 peaks that had glycoform distributions similar to the main variant, including proteoforms with one aglycosylated heavy chain. The deconvoluted spectrum of the acidic-1 variant, when overlaid with the main variant, showed the increase in intensity of the glycation modification, which was previously observed in the acidic side of the main variant.^45^ This increase in the deconvoluted intensity of A2G0F/A2G1F or A2G0F/GA20F+Hex as seen in Figure 3C again confirmed that glycation modification is enriched in the acidic variant compared to the main variant. The deconvoluted spectrum also displayed a mass shift of +1 Da in acidic peak 1 as seen in Figure 3A on the major glycoform A2G0F/A2G0F compared to the main variant indicating presence of deamidated species. This is consistent with previous finding on mAbs wherein deamidation decreased the pI ∼0.07 unit and caused a mass shift of +0.98 Da.^46,47^ Figure 3B shows the deconvoluted spectrum of acidic peak 2, which has a +4 Da shift compared to the main peak as a consequence of the combination of deamidation and potential reduced thiols. With the charge-based separation in the frontend before MS detection, deamidation can be identified on the intact level due to the distinct acidic shift from asparagine to aspartic acid. The presence of the second acidic variant can be indicative of deamidation taking place at 2 different asparagine (Asp) residue.

**Figure 3:**
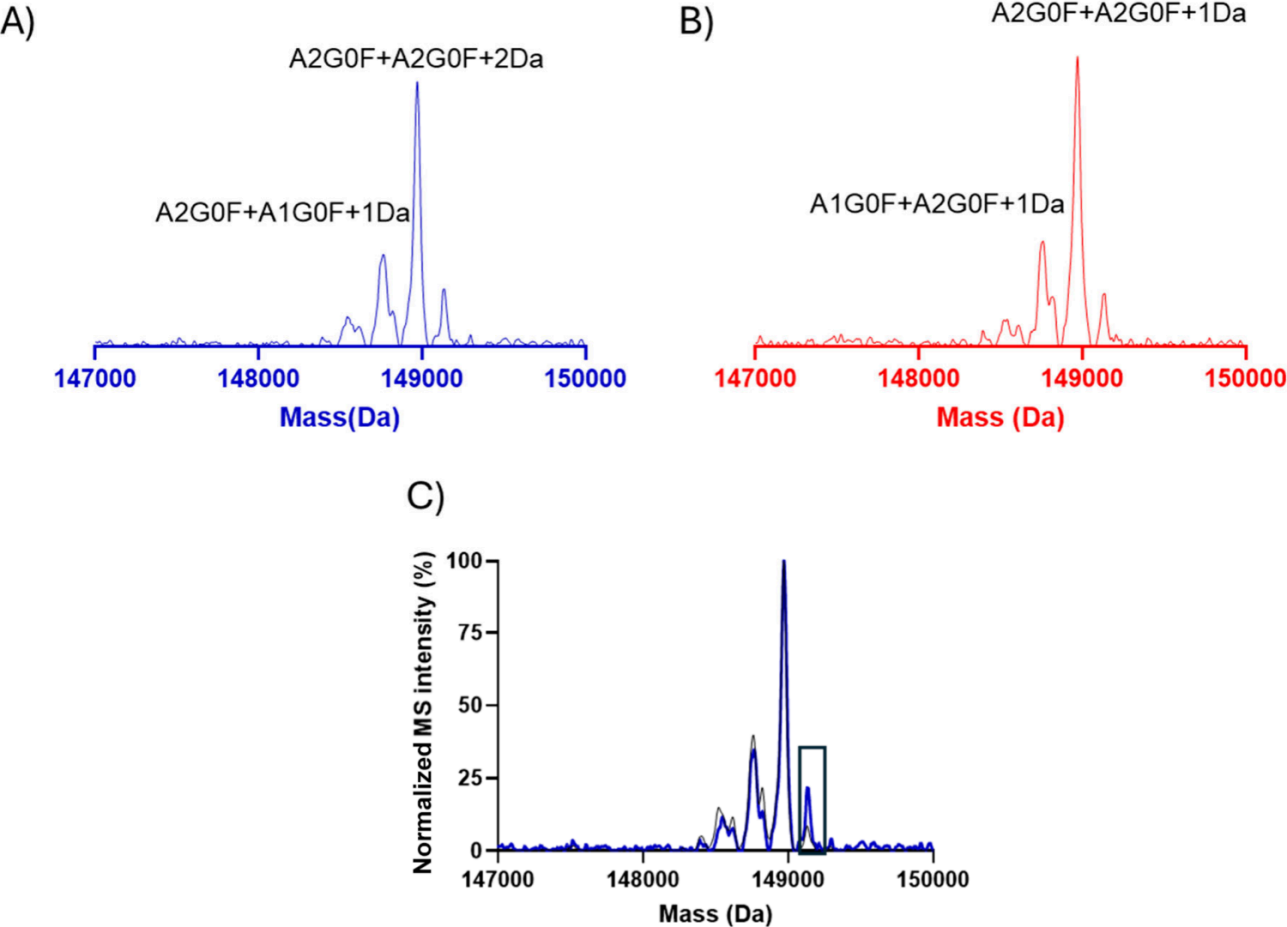
Deconvoluted spectra of acidic variants (as seen in Figure 2A) from icIEF-UV/MS analysis of mAb-1 with Intabio ZT system. (A and B) Deconvoluted spectra of the acidic variants displaying glycoforms with +2 and +1 deamidation modifications. (C) Overlay of deconvoluted spectra of the main variant (black) and the acidic variant (blue) highlighting an increase in glycation for one of the glycoforms, indicating that glycation events are enriched in the acidic variant.

Investigating the impact of various stress conditions on mAbs is crucial for identifying potential PTMs that can arise from environmental or storage-related stresses. In this study, we degraded mAb-1 at elevated temperatures and subsequently analyzed it by using icIEF-UV/MS analysis. As shown in Figure 4A, we observed an increase in acidic variants compared to the reference material wherein 4 acidic peaks were identified compared to 2 acidic peaks in the reference material. Deconvolution revealed that this increase in acidic variants was associated with enhanced deamidation, likely occurring at one or more aspartic acid residues, as illustrated in Figure 4C. Additionally, the basic variant showed a mass decrease of −16 Da on the major glycoform of mAb-1 as seen in Figure 4B, suggesting possible succinimide formation, i.e., the intermediate of the deamidation process. This succinimide modification may go undetected in peptide mapping experiments, as sample preparations are often conducted at neutral to basic pH, where succinimide would typically convert to isoaspartic acid or aspartic acid, thus obscuring its detection.

**Figure 4:**
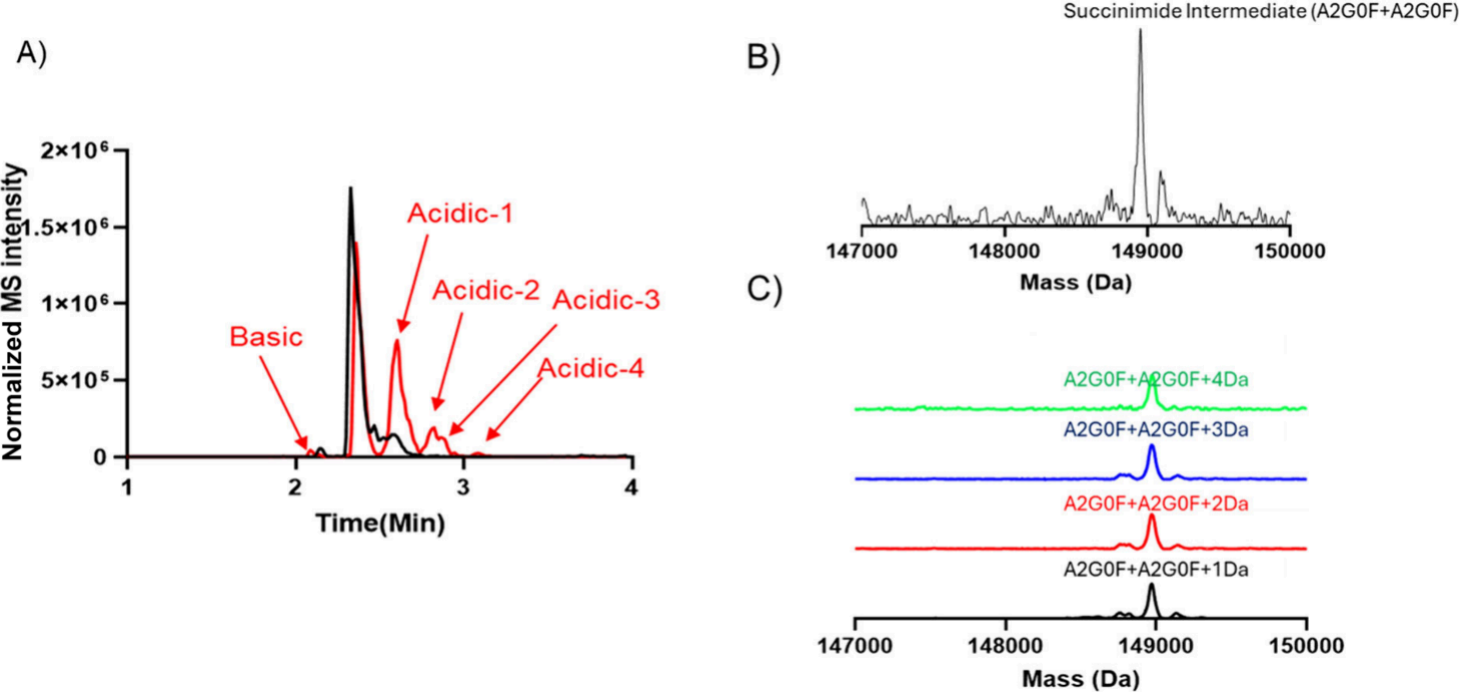
icIEF-UV/MS analysis of thermally stressed mAb-1 analyzed with the Intabio ZT system. (A) Overlay of icIEF-MS profiles of unstressed mAb-1 (black) and thermally stressed mAb-1 (red), showing an increase in acidic peaks in the thermally stressed sample. (B) Deconvoluted spectrum of the basic variant, indicating the presence of succinimide intermediate. (C) Deconvoluted spectra of distinct peaks in the acidic variant, demonstrating the presence of deamidation.

### Purification/Enrichment and Characterization of Charge Variants with Traditional Workflow

The mAb-1 was fractionated using cation exchange high-performance liquid chromatography (CEX-HPLC), and the collected fractions were subsequently analyzed by icIEF to verify the enrichment of acidic, main, and basic variants. The identified fractions were pooled and buffer-exchanged into the formulation buffer, followed by further purification and enrichment assessment using the optimized icIEF method.

The resulting icIEF profiles, along with the purity and enrichment data of the isolated fractions, as shown in Figure S2 and Table S1, demonstrate a significant enrichment of charge variants relative to the original mAb-1. This heightened enrichment allows for more robust characterization of PTMs associated with each charge variant. These PTMs were comprehensively analyzed through multiple analytical techniques, including SEC, reduced capillary electrophoresis (R-CE-SDS), and LC-based intact mass analysis. The results obtained from these analyses were comparable to the results from the Intabio ZT system.

Size heterogeneity related to enriched charge variants of mAb-1 was monitored via size exclusion high-performance liquid chromatography (SEC-HPLC). This technique, performed under nondenaturing conditions, resolves both HMW and LMW species from the main peak, which primarily represents the monomers. SEC-HPLC achieves separation based on differences in the hydrodynamic radius of the molecules. For mAb-1, structural heterogeneity and other modifications impacting the hydrodynamic volume were assessed.

The SEC-HPLC chromatogram for each charge variant and % peak areas can be observed in Figure 5A, revealing the presence of HMW species in the basic fraction, whereas no HMW species were observed in the enriched main and acidic fractions. This confirms with previous studies performed on other mAbs, that aggregate or HMW are seen in basic variants due to larger hydrodynamic volume related to size, resulting in higher charge and higher pI values. Although high molecular weight (HMW) species were not observed in the basic variant during Intabio ZT analysis, this absence can likely be attributed to the *m*/*z* range (2200–4400) used for icIEF-UV/MS analysis. HMW species, typically forming at *m*/*z* values exceeding 5000, would have been excluded from the analyzed spectrum.

**Figure 5:**
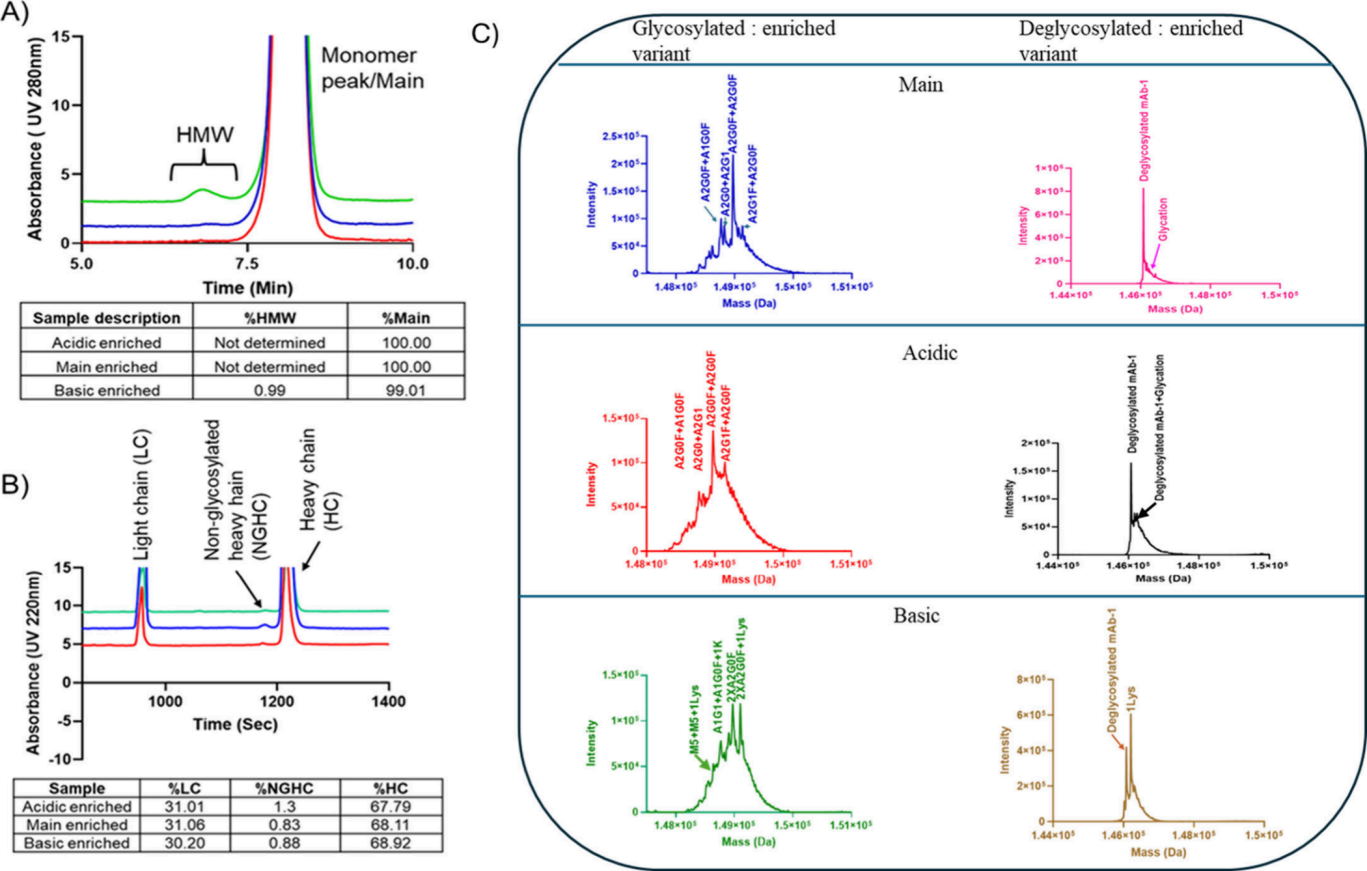
Characterization of mAb-1 using a fractionation-based workflow. (A) Zoomed-in chromatographic overlay of size exclusion chromatography profiles for the acidic variant (red), main variant (blue), and basic variant (green), with a table of percent peak areas showing the enrichment of high molecular weight (HMW) species in the basic variant. (B) Zoomed-in electropherogram overlay of reduced capillary electrophoresis profiles for the acidic variant (red), main variant (blue), and basic variant (green), along with a table of percent peak areas indicating enrichment of nonglycosylated heavy chain (NGHC) in the acidic variant. (C) Deconvoluted masses of enriched variants glycosylated and deglycosylated, displaying similar glycoforms and glycation patterns as observed in the icIEF-UV/MS analysis from the Intabio ZT system.

Peptide bond hydrolysis can result in the formation of different subunits in monoclonal antibodies, potentially affecting the product quality. Reduced (R-CE-SDS) spectroscopy was applied to mAb-1 to characterize size-based variants. Separation in this assay is inversely proportional to hydrodynamic volume, enabling highly resolved separation of size variants and PTMs, such as nonglycosylated heavy chains and other size-based variants that are smaller than the light chain, larger than the heavy chain, or have masses between those of the heavy and light chains. R-CE-SDS profiles indicated a slight increase in the nonglycosylated heavy chain (NGHC) content was observed in the fractionated enriched acidic variant compared to other enriched variants, as seen in Figure 5B. Intabio analysis revealed aglycosylated heavy chain mainly in the main variant. The presence of NGHC in fractionated variants can therefore be attributed to residual main variant in the enriched charge variants, resulting from the fractionation process, as demonstrated in Figure S1. Comparing data obtained from the Intabio ZT analysis helps mitigate biases that may arise from the fractionation process, thereby providing a more accurate representation of modifications associated with biotherapeutics.

Intact mass analysis of mAb-1 was conducted using liquid chromatography coupled to mass spectrometry (LC-MS). This analysis provided data on glycan distribution and identified PTMs under glycosylated and deglycosylated conditions. As seen in Figure 5C, the deconvoluted mass spectra of main and acidic fractions revealed a consistent G0F/G0F glycoform across these variants as well as the presence of glycation, which was observed in main and acidic variants under deglycosylation conditions. Corroborating the findings from the Intabio ZT system, unprocessed C-terminal lysine enrichment was observed in the basic fraction. Intact mass analysis provided valuable insights into major modifications, corroborating the results obtained from the Intabio ZT analysis. However, low-abundance modifications, such as the presence of two C-terminal lysine residues and other potential oxidation modifications, were not detected by using this method. This limitation may be attributed to the fractionation process, wherein residual charge variants were observed in the enriched fraction resulting in hindrance of identification of modifications.

## Discussion and Conclusions

The comparative performance between icIEF-UV/MS and the traditional workflow successfully demonstrated comparable results. The Intabio ZT system effectively characterized charge variant peaks and modifications including glycation, deamidation, C-terminal lysine variants, oxidation, and the distribution of glycoforms in mAb-1 samples. The comparable icIEF electropherograms generated by the Maurice system and the icIEF-UV profiles obtained using the Intabio ZT platform enable confident and precise assignment of post-translational modifications (PTMs) across various charge variants of mAb-1. This alignment not only validates the reliability of the new workflow but also underscores its value as a rapid, unbiased, and high-resolution approach for comprehensive charge variant characterization in biotherapeutic development. The developed workflows also demonstrate utility in rapidly characterizing degradation pathways, as evidenced by the analysis of thermally stressed mAb-1 samples.

Characterization of mAb-1 using traditional fractionation required multiple injections to collect sufficient material for various analytical assays. In contrast, icIEF-UV/MS provided equivalent information from a single injection and a 30 min acquisition. Additionally, the icIEF-UV/MS approach enabled the identification of modifications associated with individual peaks in the acidic region, whereas the traditional approach could identify only modifications associated with the overall population of acidic variants.

In conclusion, the comparative evaluation of icIEF-UV/MS and conventional charge variant characterization workflows highlight the distinct advantages of integrating mass spectrometry with capillary isoelectric focusing. The icIEF-UV/MS platform not only streamlines analytical timelines but also significantly improves the resolution and characterization of post-translational modifications (PTMs), offering a more comprehensive and efficient approach to profiling charge heterogeneity in biologics. As the biologics industry advances, the implementation of cutting-edge analytical techniques such as icIEF-UV/MS across diverse biotherapeutic modalities will be pivotal for the rapid, detailed, and unbiased characterization of novel protein-based therapeutics. Such integration is essential for meeting regulatory expectations regarding the precise characterization of therapeutic candidates, ultimately supporting the safety and efficacy of antibody-based treatments.

## Supplementary Material



## Notes

Additional data supporting the comparative assessment of icIEF-UV/MS and fractionation workflows for antibody charge variant analysis, spectra showing glycation-related shifts, electropherogram overlays highlighting variant enrichment, and quantitative peak area data from fractionated samples (PDF)
