# Supplementary material for: Accelerating Characterization of Therapeutic Antibodies: A Comparative Assessment of icIEF-UV/MS and the Traditional Fractionation Workflow
Source: J Am Soc Mass Spectrom. 2025 Jul 28;36(8):1641–9. doi: 10.1021/jasms.5c00058 (PMC12333362; doi:10.1021/jasms.5c00058)
Supplement: Supplementary file 1 [file js5c00058_si_001.pdf]

Supplementary Information

Accelerating characterization of therapeutic antibodies: A comparative assessment of icIEF-UV/MS and traditional fractionation workflow

Authors: Arnik Shah[a*], Parth Shah[a], Alex Johnson[a], Jean Bender[a], Dmitry Gumerov[a], Jingwen Ding[b], Scott Mack[b], Matthew D. Stone[b], Maggie A. Ostrowski[b]

Affiliations: [a]*Visterra Inc, 275 2nd avenue, Waltham, MA-02451, United states*

[b] *SCIEX, Fremont, CA-94538, United states*

Corresponding author email: ashah@visterrainc.com

Table of Contents.

**Figure S1:** Relative intensities of deconvoluted spectra for the main peak along with its acidic pI shifts.	S-2
**Figure S2:** Charge variant enrichment from fractionation-based workflow	S-2
**Table S1:** %Peak areas of enriched charge variant from fractionation-based workflows.	S-2

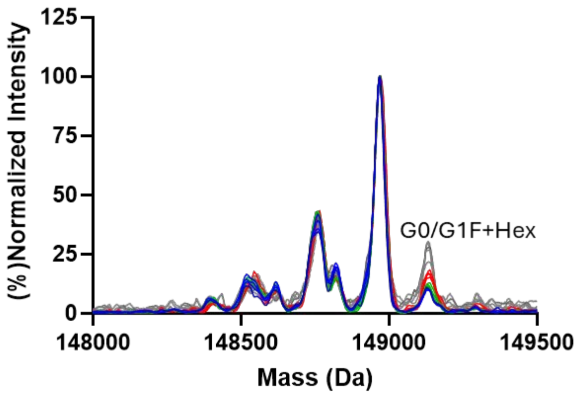

Figure S1: Relative intensities of deconvoluted spectrum in main peak along the acidic pI shift showing increase in intensity of G0/G1F species indicating presence of glycation as modification in main species.

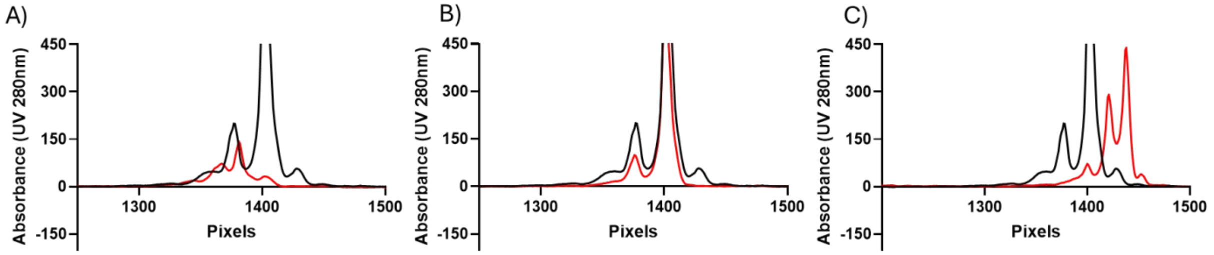

Figure S2: Charge variant enrichment from fractionation- based workflow. (A) Electropherogram overlay of enriched acidic (red) and reference mAb-1(black). (B) Electropherogram overlay of enriched main (red) and reference mAb-1(black). (C) Electropherogram overlay of enriched Basic (red) and reference mAb-1(black).

Table 1: %Peak areas of enriched charge variant from fractionation-based workflows.

**Sample Description**	%Acidic	%Main	% Basic
Enriched Acidic	64.47	31.72	3.82
Enriched Main	20.96	79.04	0.13
Enriched Basic	11.91	36.29	51.80
